# Characterization of the Micro‐Morphology and Compositional Distribution of Chang'e‐5 Lunar Soil Mineral Surfaces Using TOF‐SIMS

**DOI:** 10.1002/advs.202416639

**Published:** 2025-01-22

**Authors:** Tinglu Song, Jie Liu, Chunlin Zhang, Xinyu Yang, Taiyang Chen, Shunzi Jiang, Fan Xu, Ning Li, Menghua Zhu, Shaolin Li, Meishuai Zou

**Affiliations:** ^1^ Experimental Center of Advanced Materials School of Materials Science & Engineering, Beijing Institute of Technology Beijing 100081 China; ^2^ LandSpace Technology Co., Ltd. Beijing 100176 China; ^3^ School of Electronics and Communication Engineering Sun Yat‐sen University Shenzhen 528406 China; ^4^ State Key Laboratory for Artificial Microstructure and Mesoscopic Physics School of Physics Frontiers Science Center for Nano‐optoelectronics & Collaborative Innovation Center of Quantum Matter Peking University Beijing 100871 China; ^5^ State Key Laboratory of Lunar and Planetary Sciences Macau University of Science and Technology Macau 519020 China

**Keywords:** chang'e‐5, compositional distribution, lunar soil, mineral, TOF‐SIMS, vesicle

## Abstract

The lunar soil samples returned by China's Chang'e‐5 (CE‐5) contain valuable information on geological evolutions on the Moon. Herein, by employing high‐resolution time‐of‐flight secondary ion mass spectrometry (TOF‐SIMS), five rock chip samples from the CE‐5 lunar soil are characterized in‐depth, which reveal micro‐morphological and compositional features. From the elemental/molecular ion distribution images, minerals such as pyroxene, ilmenite, feldspar, K‐rich glass, silica, and silicate minerals are identified, along with their occurrence states and distribution results. More importantly, uncommon vesicle‐like patterns are probed via TOF‐SIMS, which may not be captured by conventional electron microscopy. The possible origins of vesicles are also proposed. Hopefully, these discoveries will provide essential guidance for future investigations on the Moon and accelerate the application of TOF‐SIMS in space exploration.

## Graphical abstract

Lunar soil samples from Chang'e 5 are characterized by high‐resolution time‐of‐flight secondary ion mass spectrometry, which revealed uncommon vesicle patterns that may not be observed via the conventional scanning electron microscope with energy dispersive X‐ray.

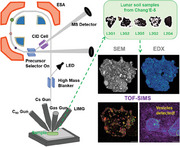

## 1 Introduction

In recent years, explorations on the Moon have attracted much attention from the broad space research community. Investigations on the Moon could promote the understanding of the origin and historical evolution of planets (e.g., Earth, Moon), as well as facilitate future exploitation of the abundant potential resources on the Moon. Therefore, since the 1960s, space exploration on the Moon has been initiated and developed rapidly. During this period, orbital probes captured and completed high‐definition images of the lunar surface and the first comprehensive photographic atlas of the Moon. However, it is far from satisfactory to roughly probe the surface of the Moon rather than obtain in‐depth information from its on‐site samples, i.e., lunar soils, which contain essential information on the long‐term formation and evolution of the Moon. Characterizations on the lithology,^[^ ^1^ ^]^ chemical composition,^[^ ^2^ ^]^ impact flux,^[^ ^3^ ^]^ and forming age^[^ ^4^, ^5^ ^]^ of unknown lunar regions of lunar soil samples provide valuable insights toward elucidating the rock diversity of the lunar crust,^[^ ^6^ ^]^ impact processes^[^ ^7^, ^8^ ^]^ on the lunar surface and the evolutionary history of the Moon.

However, further investigations on lunar soil were significantly limited by the sample scarcity. Moreover, the returned samples were obtained from specific regions, which may not represent the broad lunar surface features. More importantly, lunar sampling has experienced a gap of more than 40 years^[^ ^9^ ^]^ since the United States conducted six lunar sampling missions between 1967 and 1972, and the Soviet Union conducted three lunar sampling missions between 1970 and 1976. Inspiringly, as China's first unmanned lunar sample‐return mission, Chang'e‐5 (CE‐5) landed its probe in the Procellarum Basin in the northeastern part of the Moon (43.06°N, 51.92°W)^[^ ^10^ ^]^ on December 1, 2020, and on December 17, 2020, successfully returned 1.731 kg of lunar soil,^[^ ^11^ ^]^ which has been recognized as the world's largest single sampling volume and highest sampling latitude for unmanned lunar sampling missions to date. Statistical dating of lunar impact craters indicates that the Lunar Sea Unit in the CE‐5 landing area is relatively young,^[^ ^12^ ^]^ implying that the returned CE‐5 lunar soil samples may carry information about the youngest volcanic activity on the Moon, which provides new opportunities to unravel the missing information about the Moon. In addition, previous studies on CE‐5 loam mainly focused on basalt,^[^ ^13^, ^14^, ^15^, ^16^, ^17^, ^18^ ^]^ while ignoring several important types of lunar crustal rocks such as breccia^[^ ^19^ ^]^ and other types of magmatic rocks, which are imperative to be investigated.

Apart from sample limitations, related investigations on the structure, composition, and intrinsic properties of lunar soil are also confined by conventional characterization methods, which require more comprehensive and in‐depth measurements. In recent years, time‐of‐flight secondary ion mass spectrometry (TOF‐SIMS) has emerged as a powerful tool for lunar soil investigations,^[^ ^20^, ^21^ ^]^ which exhibits excellent element sensitivity, high spatial resolution, low detection limit, and non‐destructive probing features.^[^ ^22^, ^23^, ^24^ ^]^ The essential working principle of TOF‐SIMS is that the sample surface will be first bombarded by a pulsed primary ion beam (e.g., Ga^+^, Bi^3++^, etc.) under high vacuum conditions, after which various secondary ions including both positive and negative species with different m/z values will be generated and collected into the detector by adding high voltage potential. Their respective ion mass will then be determined by measuring their time of flight from the sample to the detector. In particular, TOF‐SIMS could acquire in situ ion mapping images with high lateral resolution,^[^ ^25^, ^26^, ^27^ ^]^ which is beneficial for identifying unknown mineral particles or sub‐particles,^[^ ^28^, ^29^ ^]^ understanding the distribution of various ions inside the sample,^[^ ^30^ ^]^ as well as obtaining essential information about the original diagenetic environment of cosmic samples. Due to the above advantages, TOF‐SIMS has been widely applied to characterize and analyze certain interplanetary^[^ ^31^ ^]^ and pre‐solar system dust particles.^[^ ^32^ ^]^ For instance, Leitner et al used TOF‐SIMS to reveal that comet particles in the Stardust mission impacted the Aluminum foil residues are closely related to polycyclic aromatic hydrocarbons (PAHs),^[^ ^33^ ^]^ which not only provides new information for understanding the material composition and evolution of comets but also reflects that TOF‐SIMS has great potential for cosmic sample detection. Nevertheless, there is still a lack in‐depth investigation on lunar soil samples by TOF SIMS.

Herein, we reveal in‐depth the lithological, compositional, and structural information of five distinct lunar soil samples (including magmatic rock and breccia) returned from CE‐5 through TOF‐SIMS measurements. The morphologies of each lunar sample were first probed and compared via electron microscopy.^[^ ^34^ ^]^ Then, the chemical compositions, end‐member components, and minute inclusions of the lunar soil samples were revealed by TOF‐SIMS. Moreover, relevant petro‐mineralogical and geochemical analyses were carried out to better understand the composition and structure of individual matrix particles, as well as the potential relationship between different mineral particles, thus providing essential information for future Moon scientific investigations. In addition, to make a comparison, scanning electron microscopy with energy dispersive X‐ray (SEM‐EDX) measurements were also conducted, which exhibit similar functions as TOF‐SIMS such as acquiring compositional and morphological information. Importantly, vesicles could only be observed by TOF‐SIMS, instead of the SEM‐EDX method. These findings highlight the significance and necessity of TOF‐SIMS in lunar‐based studies.

## 2 Experimental Section

### 2.1 Sample Preparation

**Figure 1:**
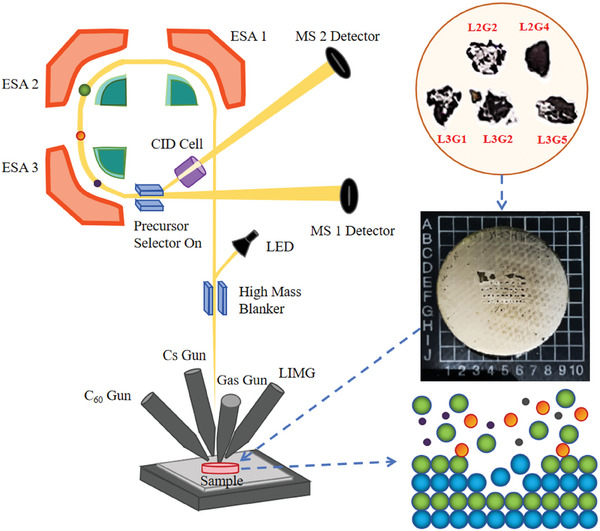
Schematic illustration of the working principle of employing TOF‐SIMS to characterize lunar soil samples.

Five typical debris samples were selected from lunar soil samples (No. CE5C0400 and No. CE5Z0403YJYX012), including three magmatic rocks (No. L2G4, No. L3G2, No. L3G5) and two breccias (No. L2G2, No. L3G1). In this work, lunar soil samples were named based on their positions on the epoxy mounts. For example, L2G4 and L3G5 refer to samples in row 2, column 4, and row 3, column 5, respectively. Each lunar soil fragment was 250–500 µm in size. The debris samples were mounted on 1‐inch round epoxy mounts and polished using adamantine gypsum (9, 3, and 1 µm) and an alumina suspension (0.05 µm) (**Figure** **1**).

### 2.2 Characterizations

The backscattered (BSE) images were acquired by a Japanese electronic IT‐500 tungsten filament scanning electron microscope (SEM). Equipped with the Ultim Max170 energy‐dispersive spectrometer, the SEM facilitated mineral identification and enabled the acquisition of X‐ray elemental mappings at a voltage of 15 kV. Carbon was sprayed onto the sample surface for further SEM measurements.

TOF‐SIMS measurements were performed using a PHI nano ToF II (ULVAC PHI Inc., Chigasaki, Kanagawa, Japan), which used a Bi_3_^++^ beam current (30 kV) as the main beam current for sample detection. Since TOF‐SIMS was conducted after SEM measurements, the sample surface has already been covered by carbon. Therefore, before collecting SIMS signals, only selected sample regions (≈1.0 mm^2^) were sputtered with an Ar ion beam, while the rest sample surfaces were still covered by carbon. As a result, no additional charge compensation was required for TOF‐SIMS measurements. To obtain a higher spatial resolution, the unraveling beam mode (UB mode) was also used. The beam size under the normal and UB modes was set as 1000 and 100 nm, respectively. A decreased beam size is beneficial for improving the spatial resolution of the measured figures. The scanning pixel in both modes is 512 × 512 pixels. The DC beam current in normal and UB modes is 2 nA and 200 pA, respectively. The data collection time for normal and UB modes is 3 and 6 min, respectively.

## 3 Results and Discussion

### 3.1 Lunar Magmatic Rock Samples

#### 3.1.1 L3G5

**Figure 2:**
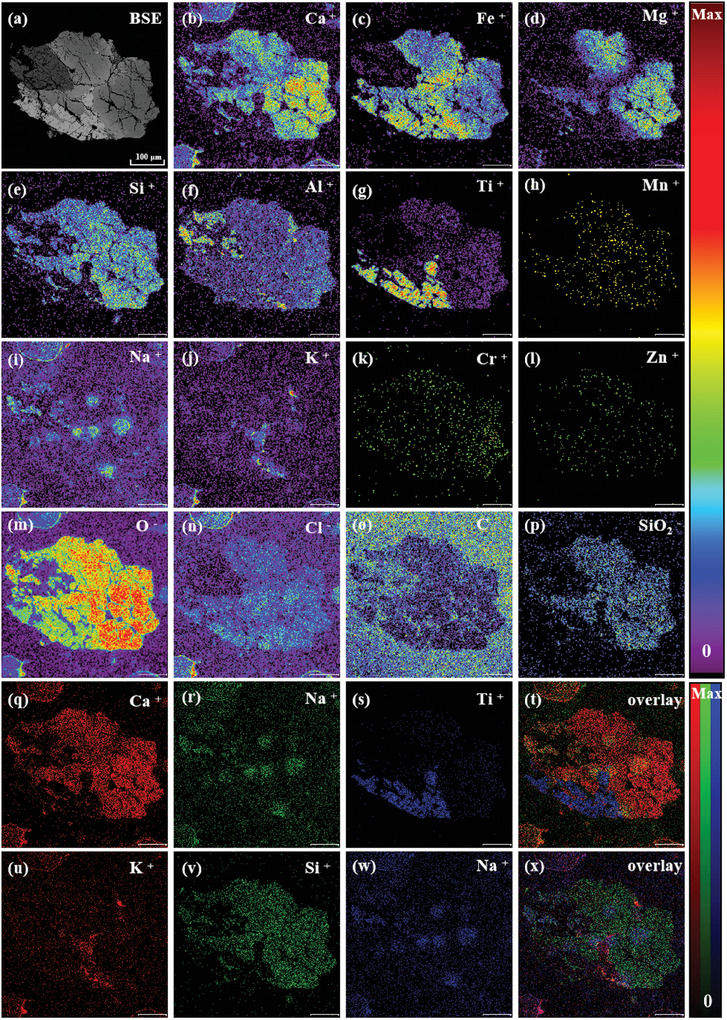
TOF‐SIMS mapping images of the L3G5 sample under UB mode (scale bar is 100 µm).

BSE imaging shows that L3G5 exhibits a coarse grain size, and more developed fractures, and its constituent minerals have obvious compositional zoning (**Figure** **2**a). Then, TOF‐SIMS measurements were conducted to reveal the surface features of the lunar soil sample. The 2D imaging results are illustrated in Figure 2. The color bars on the right side indicate the degree of the ion signal intensity. The intensity increases with the bar color changed from black to red, where Max and 0 refer to the highest and lowest ion intensity, respectively. The TOF‐SIMS secondary ion image results also show that L3G5 has obvious block characteristics (Figure 2b–p), indicating that it is composed of multiple minerals. Among them, Fe, Ti, and O are enriched and distributed in a columnar manner in the same micro‐area (≈100 µm) in the lower right corner of the entire crystal. Only a small amount of Al is detected in this area, without the presence of Ca, Si, and Mg, suggesting that the light gray crystals here are mainly ilmenite (Figure 2q–t). Ti is mostly distributed in K‐ and Si‐poor regions, indicating barely any K‐ and Si‐rich inclusions (e.g., SiO_2_) in the ilmenite crystal. The distribution of Mg and SiO_2_ shows an obvious correlation and zoning characteristics. These regions are enriched in Ca, Fe, Si, and Al to varying degrees. In addition, local Na and K aggregates were found, which indicates that these detrital crystals are likely to be composed of a mixture of silica and feldspar. Among them, Ca is mostly enriched in the large‐grained crystals on the right, and there is obvious Fe enrichment at the junction of the detrital crystal blank, which is in accordance with the compositional characteristics of pyroxene. As shown in Figure 2u–x and K is enriched in several fine particles in the main crystal phase, which contain almost no Si, Ca, Na, and Mg, and may belong to the K‐rich mineral type.

**Figure 3:**
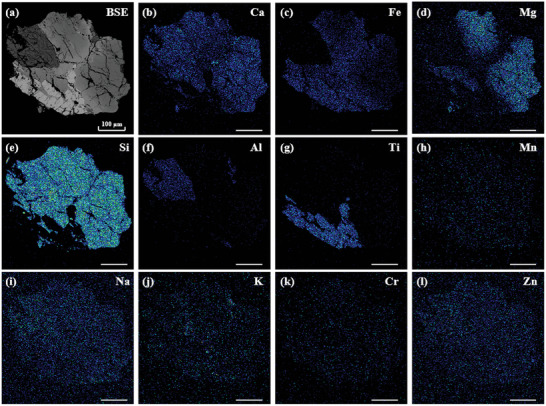
EDX images of the L3G5 sample (scale bar is 100 µm).

Specifically, there are vesicular regions at the edge of the test micro area, which not only contain concentrated K, Na, Ca, and related oxides, but also comprise a small amount of rare earth elements (REE) such as La, Tm, and Pr. Importantly, there exhibits no vesicle in the EDX image of the same sample (**Figure** **3**). Comparing EDX images with TOF‐SIMS mapping images, it can be observed that the distribution of all elements/ions is almost consistent throughout the interior of the mineral. However, the distribution of elements specific to the vesicle region, such as K, Na, and Ca (Figure 3b,i,j), is significantly different in these two techniques, where EDX fails to detect these elements in vesicular regions. In addition, TOF‐SIMS could probe multiple ions such as SiO_2_^−^, while EDX may only detect a single element.

#### 3.1.2 L3G2

**Figure 4:**
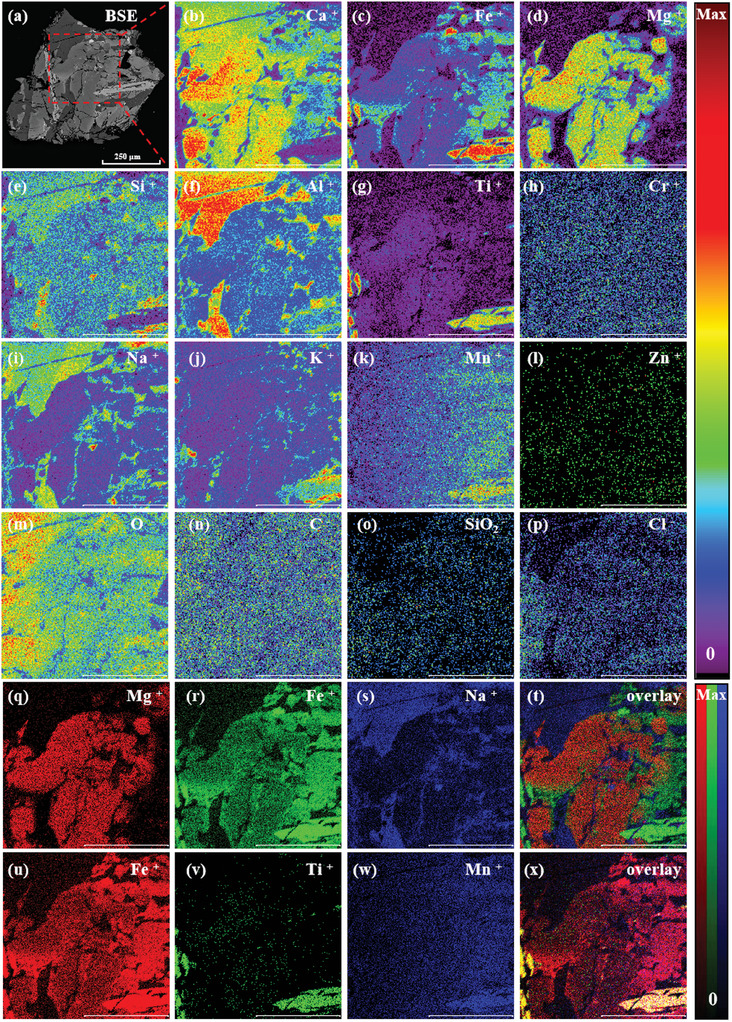
TOF‐SIMS mapping images of the L3G2 sample under UB mode (scale bar is 250 µm).

Similarly, the BSE images reveal that L3G2 mineral particles are finer, with more developed cracks, and the constituent minerals are mostly irregular in shape, with some being needle‐shaped or columnar and containing inclusions (**Figure** **4**a). To better observe the surface microscopic feature of the L3G2 sample, the region of interest (ROI) function was employed, instead of full‐scale images. The TOF‐SIMS results of the typical micro area of L3G2 show that it is composed of various types of irregular minerals (Figure 4b–p). Among them, the central part of the micro‐area is a large‐grained pyroxene columnar crystal core, with Mg enriched in the core and Fe enriched at the edges. Na, K, Si, and Al are enriched to varying degrees in the voids of Mg and Fe in large particles, which indicates the presence of feldspar‐like minerals encapsulated in the columnar pyroxene (Figure 4q–t). In addition, needle‐shaped regions with enriched Fe, Ti, and Mn in the lower right corner of the test microstructure were also observed, which suggests the presence of ilmenite (Figure 4u–x). Moreover, the peripheral area of ilmenite shows enrichment of K and Si, due presumably to the presence of K‐rich glass and SiO_2_ phases, which appear together with needle‐shaped ilmenite as interstitial phases. In addition, elemental distributions in the L3G2 sample from EDX images (Figure S1, Supporting Information) are basically identical to TOF‐SIMS.

#### 3.1.3 L2G4

**Figure 5:**
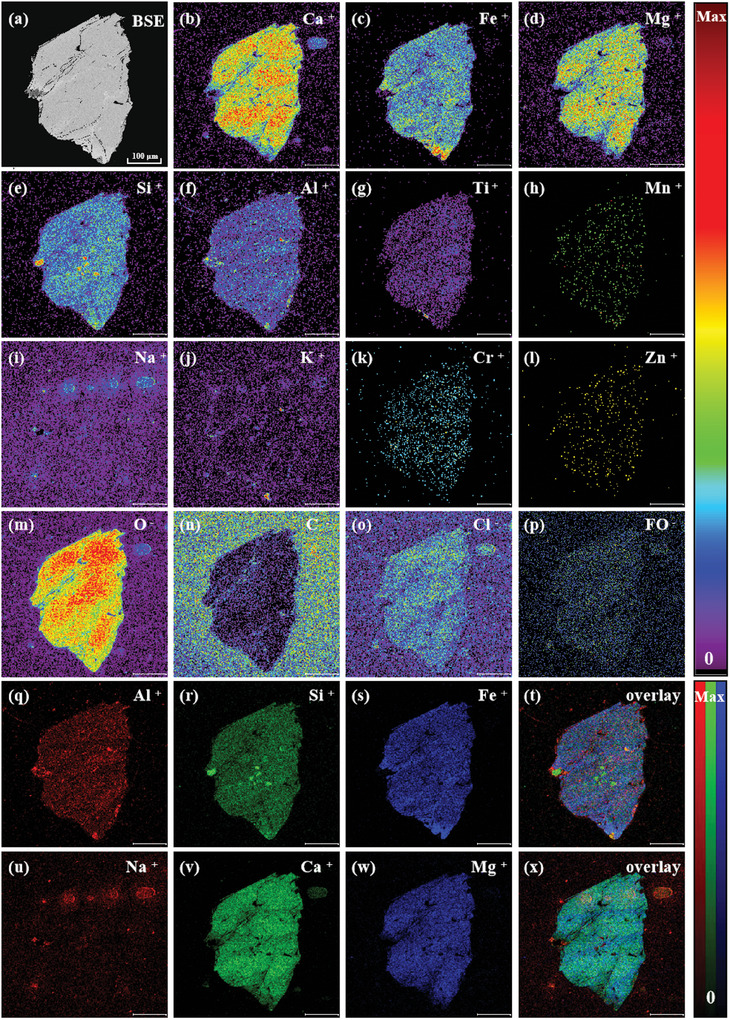
TOF‐SIMS mapping images of the L2G4 sample under UB mode (scale bar is 100 µm).

BSE imaging shows that the main body of the L2G4 debris consists of the same mineral (**Figure** **5**a), with multiple sets of non‐directional cleavages interspersed by irregular veins, where some cleavages and veins are overlapped in certain locations and one of the veins is connected to internal inclusions of the main mineral. TOF‐SIMS secondary ion images show that in the L2G4 samples (Figure 5b–p), the dominant trace elements that form the crystalline phases include Si, O, Fe, Mg, and Ca, and the trace elements are Al, Ti, Cr, Mn, and Zn. Among them, Fe and Si are characterized by banded and diffuse enrichment. The vein positions observed by BSE are consistent in the banding‐enriched areas, while the diffuse‐enriched areas are located near the internal inclusions inside the grains (Figure 5q–t). Such a phenomenon indicates that the veins interspersed in the main crystal are rich in Fe and Si. To be more specific, the inclusions are mainly Si‐rich phases, and the adjacent main crystals are rich in Fe. There is no obvious Na and K enrichment signal inside the crystals, among which Na is enriched in several small particles outside the main crystal, and K is distributed in a stripe‐like manner in the detected area. Na and K are mostly present in areas with low Si, Al, and Ca phase contents, which may be contaminated (Figure 5u–x). Combining the above results of main trace elements and related ion distributions, the L2G4 bulk mineral is categorized as pyroxene. The L2G4 sample was also measured by EDX (Figure S2, Supporting Information), which revealed similar distribution patterns as from TOF‐SIMS.

### 3.2 Lunar Breccia Samples

Compared to lunar magmatic rock samples, only a few studies on lunar breccia samples have been reported. Notably, these precious breccia samples record critical information about the impact history of the Moon's surface in a much richer and more detailed manner. It will be of great significance to investigate the multi‐source minerals within the lunar breccia for comprehensively understanding the Moon. Therefore, in this section, various types of rock samples such as lunar breccias and lunar magmatic rocks were systematically analyzed, which will facilitate the understanding of the geological evolutionary history and material compositional characteristics of the Moon.

#### 3.2.1 L3G1

**Figure 6:**
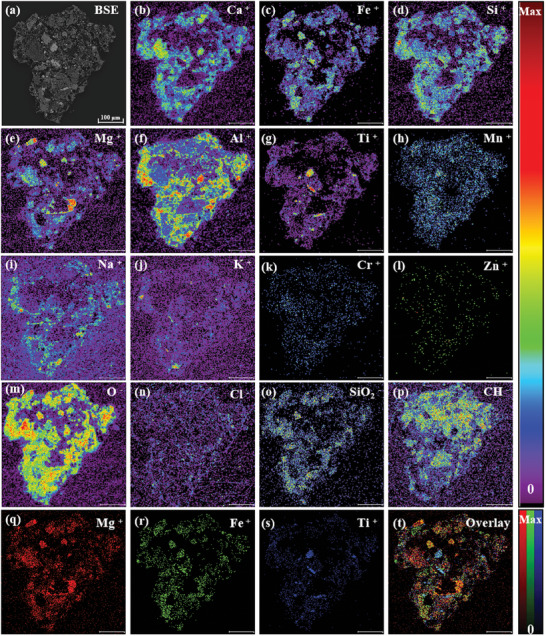
TOF‐SIMS mapping images of the L3G1 sample under UB mode (scale bar is 100 µm).

BSE result shows that L3G1 is mainly composed of basalt clasts, mineral fragments, and cemented glass. Numerous small glass spheres and air holes (<30 µm) with subcircular or elliptical shapes are observed in the clast matrix (**Figure** **6**a). TOF‐SIMS results unravel a relatively clear partitioning of different ions within the L3G1 clasts (Figure 6b–p), with most metal ions and their oxides concentrating around the periphery of the clasts and lower regions, while the upper regions are significantly enriched in hydrocarbons. To be more specific, Si, Al, Ca, Fe, and Mg are present in varying degrees within the peripheral matrix particles. The distribution of Mg within the clasts is significantly correlated with Fe (Figure 6q–t), which is primarily present in the Mg‐enriched areas, suggesting that these tiny mineral grains may be spinel. Furthermore, the Fe distribution is also obvious in Ti‐enriched areas within the rock chips, indicating that these tiny fragmented crystals are probably ilmenite. Si is enriched in the peripheral areas of ilmenite, suggesting the possible presence of the silica phase. Figure S3 (Supporting Information) illustrates the EDX results of the L3G1 sample, whose compositional distributions were closely matched to TOF‐SIMS results.

#### 3.2.2 L2G2

**Figure 7:**
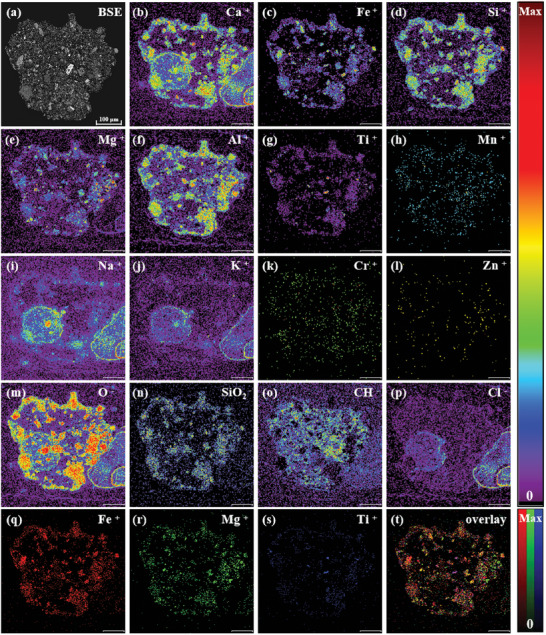
TOF‐SIMS mapping images of the L2G2 sample under UB mode (scale bar is 100 µm).

BSE imaging shows that L2G2 has a breccia structure with irregular internal debris shapes, mainly composed of fine particles, indicating that L2G2 belongs to fine‐grained detrital breccia (**Figure** **7**a). TOF‐SIMS results reveal a relatively complex ion distribution in L2G2. Most metal ions and oxides are concentrated around the periphery of the rock debris and form a ring‐like structure, while hydrocarbons are significantly enriched inside the rock debris (Figure 7b–p). The distribution of metal ions such as Ca, Fe, Si, Mg, Al, and Ti shows similar patterns within the rock debris, especially in the coarse crystal areas around the rock debris. These ions mostly exist in the form of silicate minerals, likely belonging to pyroxene debris. In detail, Fe is found in the Ti concentration area, particularly in some extremely small particles at the center of the rock debris, which may be identified as ilmenite (Figure 7q–t). Mg is enriched on the right side of the rock debris, mostly distributed as small dots. Importantly, Fe also presents in several Mg‐endowed areas, which suggests the possibility of Mg‐Fe minerals within the rock debris. In addition, the enrichment of hydrocarbons inside the rock debris might be attributed to external pollution. The slight unevenness of the polished surface caused by uneven cementation may result in residual organic matter.

**Figure 8:**
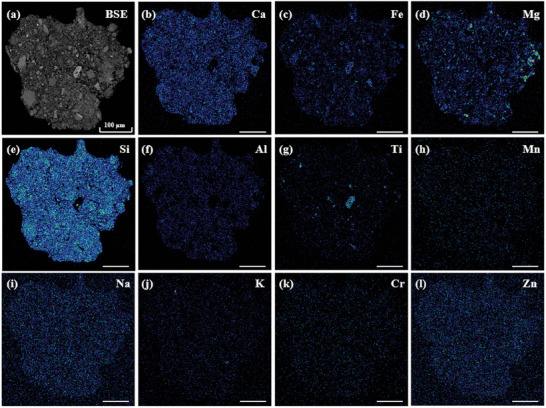
EDX images of the L2G2 sample (scale bar is 100 µm).

Similar to the L3G5 sample, vesicular regions were also detected via TOF‐SIMS in the L2G2 sample, which is enriched in K, Na, Ca, and their related oxides (**Figure** **8**b,i,j). Such vesicles are distributed both at the periphery and in the bulk of mineral crystals. Nevertheless, these vesicles are still not probed in corresponding EDX images (Figure 8).

Breccia rocks L3G1 and L2G2 both exhibit distinct detrital structures, with varying degrees of internal fractures. TOF‐SIMS images show that the ion distribution in the breccia sample is relatively complex. However, a commonality of ion distribution still exists between the two samples, i.e., most metal ions and inorganic oxides are concentrated in the peripheral areas of the rock debris, while hydrocarbons are significantly enriched within the rock debris. The mineral and debris particles within the breccia exhibit small sizes and angular shapes. In addition, cracks were probed within the mineral particles, indicating that the source rocks of such breccia have undergone strong fracturing. Moreover, there are several micrometer‐sized small glass spheres and voids in specific areas of the L3G1 breccia. The formation of small glass spheres is due possibly to the glass melt generated during high‐speed impacts or volcanic eruptions. During flight, these melts undergo rapid rotation and shape adjustment before gradually cooling and solidifying. Some melts might also collide with other mineral particles, leading to further transformation such as polymerization and bonding.

### 3.3 Vesicular Material

**Figure 9:**
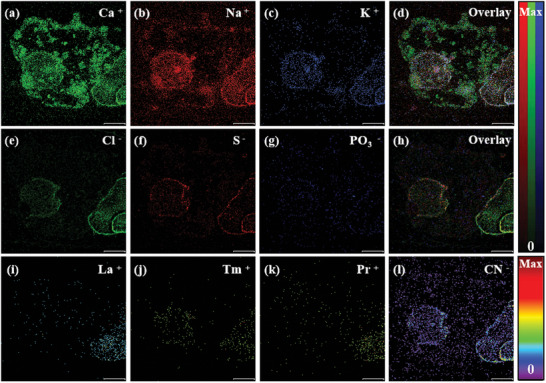
TOF‐SIMS mapping images of vesicle regions in the L2G2 sample under UB mode (scale bar is 100 µm).

It is worth noting that TOF‐SIMS detected vesicular regions in both the L3G5 and L2G2 samples, which were not found in SEM and related EDX images. To obtain SIMS mapping images, primary ions are bombarded to the sample surface and generate secondary ions. In contrast, SEM and EDX results are revealed by electrons and characterized X‐rays, respectively. In addition, the probing depth of TOF‐SIMS is within 1–2 nm, which is refined within the top surface region. Therefore, distinct signal sources and probing depths might together contribute to various signal responses of vesicles in lunar soils. Moreover, previous investigation indicates that sample properties including crystallographic orientation may also affect the collected ion intensities.^[^ ^22^ ^]^ Nevertheless, further in‐depth investigation is highly anticipated to reveal the exact mechanism of such a phenomenon. For example, three vesicle regions were found in the micro area in the L2G2 sample (**Figure** **9**). One circular vesicle was embedded within the debris, while the other two elliptical vesicles, one large and one small, overlapped and were located in the lower right peripheral area of the debris. The vesicular region is rich in K, Ca, and Na, but lacks Fe, Mg, Al, and Si. Inside the rock debris, Tm is presented, and rare earth elements (REE) such as La, Tm, and Pr, as well as a trace amount of P are also revealed in the lower right corner outside the rock debris. Additionally, there exist no obvious radioactive elements. Moreover, TOF‐SIMS is also available for detecting light elements such as H, Li, and B, which may not be revealed by the EDX method. Figure S4 (Supporting Information) presents related mapping results of these three ions for all samples. Obviously, H, Li, and B were uniformly distributed in all soil samples.

To explore reasons for the uncommon phenomenon of element enrichment and distribution pattern in vesicles, we consider two possible origins for the vesicles from the Lunar sample. First, due to inappropriate operation, several micro‐vacuoles might be introduced during the sample preparation. These vesicles may have abrasive residues that fill in during the later stages of grinding and polishing, which should be detected by TOF‐SIMS. The second possibility is that the vesicles belong to the K‐REE‐P^[^ ^16^ ^]^ component, but are limited to the vesicles wrapped inside the minerals. Although TOF‐SIMS detected incompatible elements such as K, REE, and P in the vesicle area, it did not recognize any radioactive elements. Therefore, some elements may not be detected because of the extremely low content. Whether these vesicles represent the K‐REE‐P component still requires further verification. On one hand, the vesicles outside the mineral crystal are more consistent with the first possibility. They may be affected by various factors such as the height and uniformity of the sample, resulting in resin or abrasive residue in the sample voids during the resin target sampling process. On the other hand, the vesicles inside the sample might represent a new component including the K‐REE‐P component.

## 4 Conclusion 

In conclusion, we employed the high‐resolution TOF‐SIMS technique to acquire critical information about element/ion distributions in various minerals found in the lunar soil samples returned by CE‐5. Based on the types, occurrence states, and elemental distributions, major phases including pyroxene, ilmenite, feldspar, K‐rich glass, silica, and silicate minerals were identified and validated. More importantly, we found that TOF‐SIMS could detect unique morphological features (e.g., vesicles) in lunar soils that may not be captured by the conventional SEM‐EDX method. Furthermore, component distribution and possible origins of such vesicles were comprehensively investigated. Hopefully, these findings could provide an essential understanding for lunar investigations, as well as facilitate further employment of TOF‐SIMS in related studies. Moreover, TOF‐SIMS has also demonstrated great potential in revealing the chemical composition, isotope distribution, as well as mineral structure of cosmic samples including interstellar dust, lunar soil minerals, and meteorites, etc., which will promote the understanding of the star formation mechanism and the origin/evolution of planetary materials. With the rapid development of deep space exploration missions, TOF‐SIMS is expected to become one of the most indispensable characterization tools in the field of interstellar research.

## Conflict of Interest

The authors declare no conflict of interest.

## Author contributions

T.S., S.L., and M.S.Z. conceived the idea and designed this work. T.S., J.L., and C.Z. wrote the draft manuscript. T.S., X.Y., and T.C. conducted the mass spectrometry data analysis. F.X., S.L., and M.S.Z. finalized the manuscript. S.J., N.L., and M.H.Z. conducted Chang'e data processing, calibration, and validation. T.S., J.L., F.X., and S.L. performed the morphology, geology, and compositional distribution data analysis. All the authors discussed the results and reviewed the manuscript.

## Supporting information



Supporting Information

## Data Availability

The data that support the findings of this study are available from the corresponding author upon reasonable request.
