# Supplementary material for: Characterization of the Micro‐Morphology and Compositional Distribution of Chang'e‐5 Lunar Soil Mineral Surfaces Using TOF‐SIMS
Source: Adv Sci (Weinh). 2025 Jan 22;12(11):2416639. doi: 10.1002/advs.202416639 (PMC11923877; doi:10.1002/advs.202416639)
Supplement: Supplementary file 1 — Supporting Information [file ADVS-12-2416639-s001.docx]

**Supplementary Information**

**Characterization of the Micro-Morphology and Compositional Distribution of Chang'e-5 Lunar Soil Mineral Surfaces Using TOF-SIMS**

Tinglu Song^1^, Jie Liu^1^, Chunlin Zhang^2^, Xinyu Yang^1^, Taiyang Chen^3^, Shunzi Jiang^1^, Fan Xu^4*^, Ning Li^1^, Menghua Zhu^5^, Shaolin Li^5*^, Meishuai Zou^1*^

^1^ Experimental Center of Advanced Materials, School of Materials Science & Engineering, Beijing Institute of Technology, Beijing, 100081, China

^2^ LandSpace Technology Co., Ltd., Beijing 100176, China

^3^ School of Electronics and Communication Engineering, Sun Yat-sen University, Shenzhen, 528406, China

^4^ State Key Laboratory for Artificial Microstructure and Mesoscopic Physics, School of Physics, Frontiers Science Center for Nano-optoelectronics & Collaborative Innovation Center of Quantum Matter, Peking University, Beijing 100871, China

^5^ State Key Laboratory of Lunar and Planetary Sciences, Macau University of Science and Technology, Macau 519020, China

Corresponding Authors: xufan@pku.edu.cn (F.X.); lisl@sstm.org.cn (S.L.); zoums@bit.edu.cn (M.Z.)


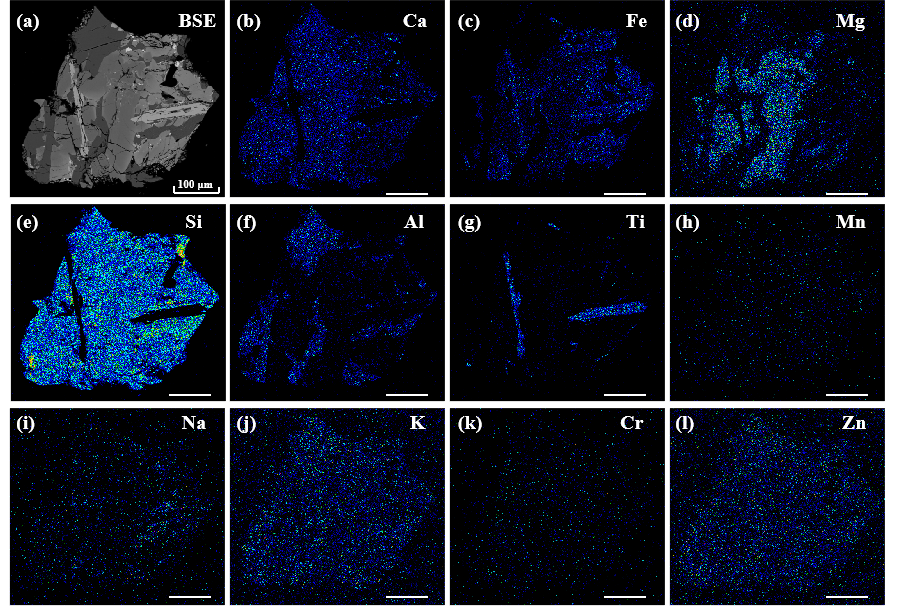


**Fig. S1.** EDX images of the L3G2 sample (scale bar is 100 μm).


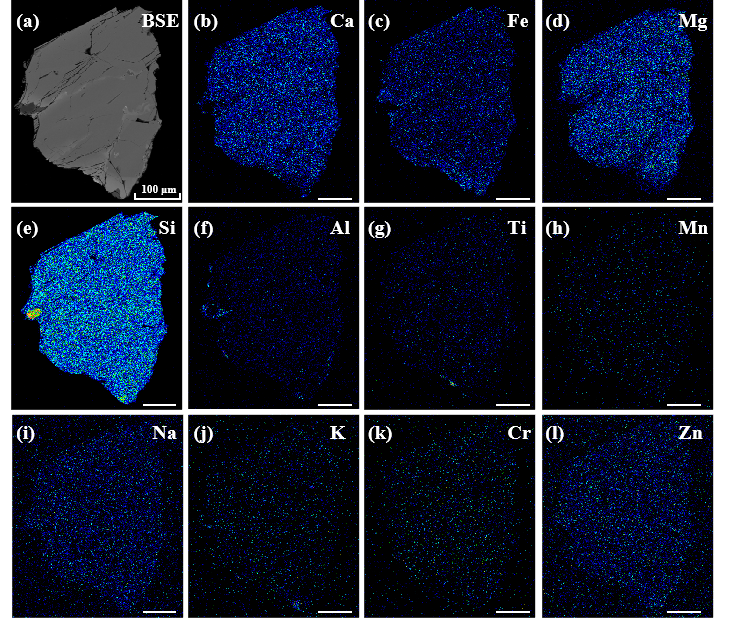


**Fig. S2.** EDX images of the L2G4 sample (scale bar is 100 μm).


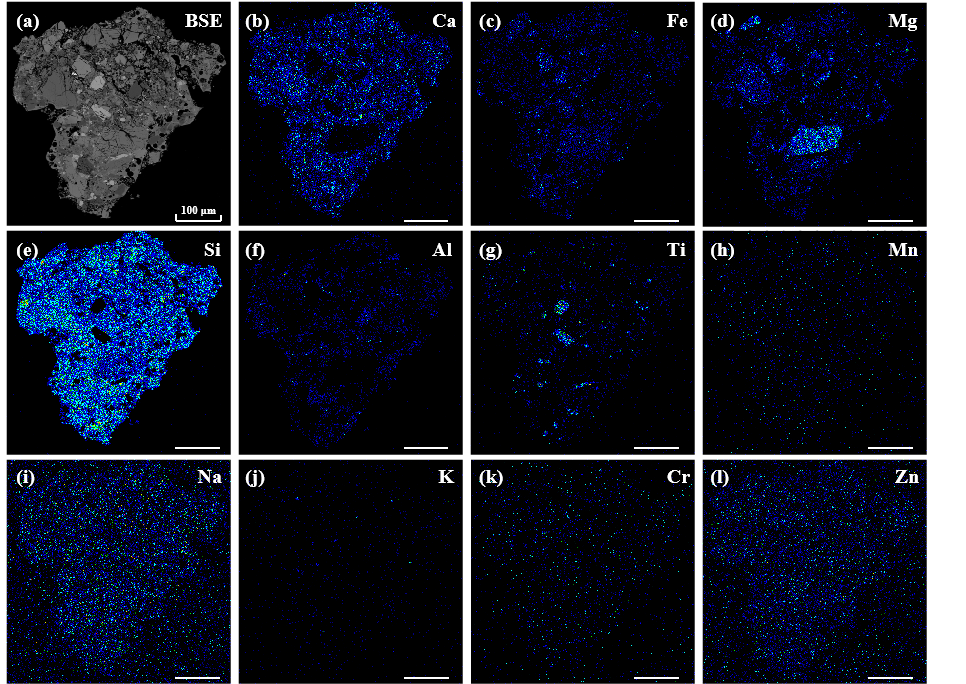


**Fig. S3.** EDX images of the L3G1 sample (scale bar is 100 μm).

**
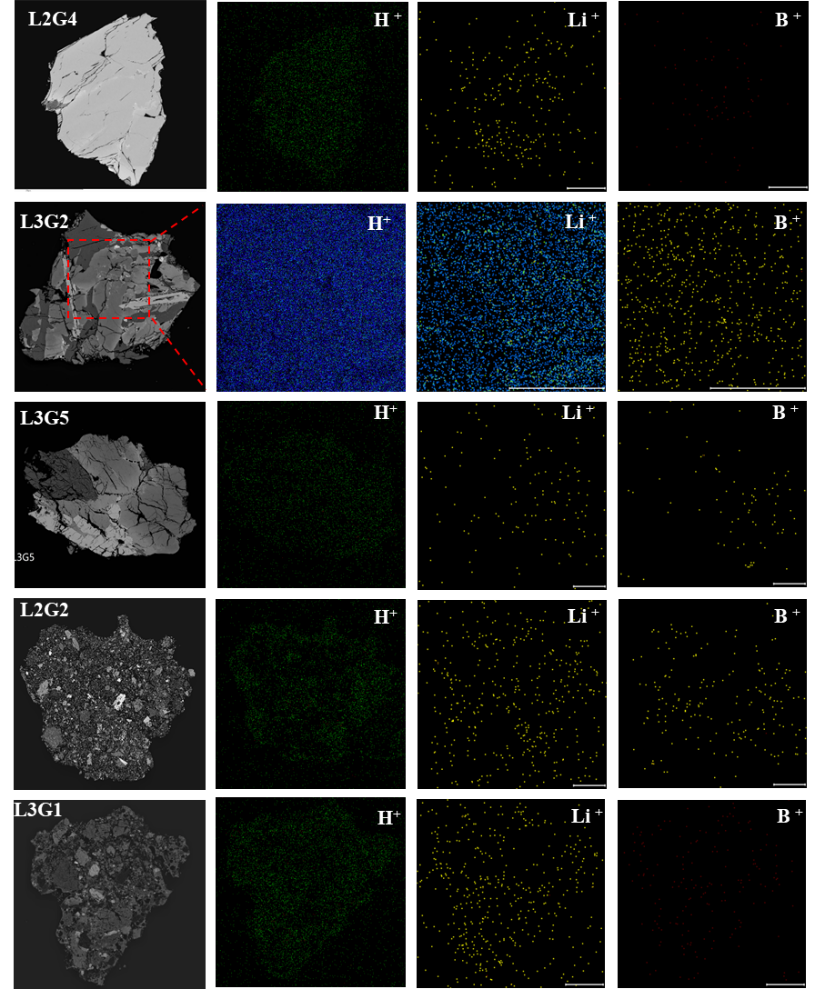
**

**Fig. S4.** TOF-SIMS mapping images of H, Li and B for all lunar soil samples.
